# Models and algorithms for genome rearrangement with positional constraints

**DOI:** 10.1186/s13015-016-0065-9

**Published:** 2016-05-17

**Authors:** Krister M. Swenson, Pijus Simonaitis, Mathieu Blanchette

**Affiliations:** LIRMM, CNRS, Université Montpellier, 161 rue Ada, 34392 Montpellier, France; Institut de Biologie Computationnelle (IBC), Montpellier, France; ENS Lyon, 46 allée d’Italie, 69364 Lyon, France; McGill Centre for Bioinformatics and School of Computer Science, McGill University, Montréal, H3C2B4 Canada

**Keywords:** Double cut and join (DCJ), Weighted genome rearrangement, Noncrossing partitions, Chromatin conformation, Hi-C

## Abstract

**Background:**

Traditionally, the merit of a rearrangement scenario between two gene orders has been measured based on a parsimony criteria alone; two scenarios with the same number of rearrangements are considered equally good. In this paper, we acknowledge that each rearrangement has a certain likelihood of occurring based on biological constraints, e.g. physical proximity of the DNA segments implicated or repetitive sequences.

**Results:**

We propose optimization problems with the objective of maximizing overall likelihood, by weighting the rearrangements. We study a binary weight function suitable to the representation of sets of genome positions that are most likely to have swapped adjacencies. We give a polynomial-time algorithm for the problem of finding a minimum weight double cut and join scenario among all minimum length scenarios. In the process we solve an optimization problem on colored noncrossing partitions, which is a generalization of the Maximum Independent Set problem on circle graphs.

**Conclusions:**

We introduce a model for weighting genome rearrangements and show that under simple yet reasonable conditions, a fundamental distance can be computed in polynomial time. This is achieved by solving a generalization of the Maximum Independent Set problem on circle graphs. Several variants of the problem are also mentioned.

## Background

A huge body of work exists on modeling the evolution of whole chromosomes [1]. The main difference between such models is the set of rearrangements that they allow. The moves of interest are usually inversion, transposition, translocation, chromosome fission and fusion, deletion, insertion, and duplication.

Almost all versions of the problem are NP-Hard if content modifying operations such at duplication, loss, and insertion are allowed [2, 3]. Fortunately, a model that considers genomes with equal content (i.e., no duplications or insertions/deletions) is quite pertinent, particularly in eukaryotes, since syntenic blocks of genes can be assigned between genomes so that each block occurs exactly once in each genome. For two genomes with equal content, double cut and join (DCJ) has been the model of choice since it elegantly includes inversion, translocation, chromosome circularization and linearization, as well as chromosome fission and fusion [4, 5].

One of the most important problems in comparative genomics is the inference of ancestral gene orders, i.e., paleogenetics. Given a realistic model of evolution, one can infer ancestral adjacencies of high confidence from present-day genomes [6–8]. However, methods that attempt to infer deeper structure for ancestral species suffer due to the huge number of parsimonious scenarios between genomes [9–11].

The apparent difficulty of the ancestral inference problem—because of the potentially astronomical number of parsimonious sorting scenarios—highlights the importance of methods that infer scenarios that conform to some extra biological constraints. Yet, aside from methods that weight inversions based on their length [12–16], to our knowledge no algorithmic work exists in this direction.

In this paper we use a weight function on rearrangements suitable for modeling *positional* constraints, i.e., sets of positions in the genome that are likely to swap adjacencies. Two examples of constraints that fit this paradigm are: (1) the physical 3D location of DNA segments in a nucleus and, (2) repetitive sequences that are the cause or consequence of rearrangement mechanisms. We illustrate the utility of our model with 3D constraints in the “Positional constraints as colored adjacencies” section.

We propose a general optimization problem that minimizes the sum of weights over the moves in a scenario. A more constrained version of the problem asks for such a scenario out of all possible unweighted parsimonious scenarios. Our algorithm solves this version of the problem in polynomial time given a binary weight function, despite an exponential growth of the number of parsimonious DCJ scenarios with respect to the distance [17, 18]. The commutation properties of DCJ moves as studied in [17] link certain DCJ scenarios to noncrossing partitions. Our algorithm relies on solving a new optimization problem on *colored* noncrossing partitions, called Minimum Noncrossing Colored Partition. It is a generalization of the Maximum Independent Set problem on circle graphs [19–21].

### Genomes as sets of signed integers

A gene, or more generally a syntenic block of genes, will be represented by a signed integer. A chromosome is a sequence of blocks, and a genome is a set of chromosomes. Thus, we write a genome in list notation where a block is a positive integer if read in one direction in the genome, and a negative integer if read in the opposite direction. For example, a genome *A* can be written as

$$\begin{aligned} \{(\circ ,5,-1,-2,6,-4,-8,\circ ), (\circ ,-3,7,\circ ), (9,10)\}, \end{aligned}$$

where $\circ$ represents a *telomere* at the end of a linear chromosome. Genome *A* has two linear chromosomes and a circular chromosome (9, 10).

Alternatively, the organization of the blocks on the chromosomes can be given by the set of adjacencies between the extremities of consecutive blocks. A block *b* has a tail extremity, written $b_t$, and a head extremity, written $b_h$. Thus, the adjacency between 5 and $-$1 in *A* is $\{5_h,1_h\}$. A block that is on the end of a linear chromosome implies a *telomeric adjacency*. The first chromosome has two such adjacencies: $\{\circ ,5_t\}$ and $\{8_t,\circ \}$. A circular chromosome has no telomeres, i.e., the last block is adjacent to the first. We can write genome *A* using adjacencies as

$$\begin{aligned} A=\big \{&\big \{\{\circ ,5_t\},\{5_h,1_h\},\{1_t,2_h\},\{2_t,6_t\},\{6_h,4_h\}, \{4_t,8_h\},\{8_t,\circ \}\big \}, \\&\big \{\{\circ ,3_h\},\{3_t,7_t\},\{7_h,\circ \}\big \}, \\&\big \{\{9_h,10_t\},\{10_h,9_t\}\big \} \big \}. \end{aligned}$$

### DCJ and sorting DCJs

Double cut and join (DCJ) is an operation on a genome that cuts one or two adjacencies, and glues the resulting ends back together according to the following rules [4]:

1. If a single adjacency is cut, then add new telomeres to the resulting ends (resulting in two new telomeric adjacencies).
2. If two adjacencies are cut, then glue the adjacencies back in one of two new ways.

Application of a single DCJ corresponds to diverse genomic operations such as inversion, chromosome linearization and circularization, transposition, and excision of a circular chromosome.

**Fig. 1:**
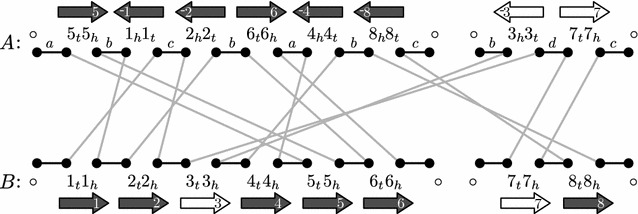
The colored adjacency graph *G*(*A*, *B*, *col*). *Black* edges are adjacency edges and *gray* edges are cross edges. The color function *col* maps adjacency edges of genome *A* to the alphabet $\{a,b,c,d\}$

The DCJ distance between genomes A and B is the minimum number of DCJ moves needed to transform A into B. DCJs that move *A* closer to *B*, called *sorting* DCJs, can be found using a graph. The *colored adjacency graph* for *A* and *B* is a graph *G*(*A*, *B*, *col*) whose vertices are the extremities and telomeres of *A* and *B*, and whose edges are colored by the color function *col*. For each adjacency in *A* or *B* an *adjacency* edge links the corresponding nodes of the adjacency, and a *cross* edge links non-telomere vertices from *A* to vertices with the same label in *B*. The graph for genomes

$$\begin{aligned} A=\big \{&\big \{\{\circ ,5_t\},\{5_h,1_h\},\{1_t,2_h\},\{2_t,6_t\},\{6_h,4_h\}, \{4_t,8_h\},\{8_t,\circ \}\big \}, \\&\big \{\{\circ ,3_h\},\{3_t,7_t\},\{7_h,\circ \}\big \} \big \}, \text {and} \\ B=\big \{&\big \{\{\circ ,1_t\},\{1_h,2_t\},\{2_h,3_t\},\{3_h,4_t\},\{4_h,5_t\}, \{5_h,6_t\},\{6_h,\circ \}\big \}, \\&\big \{\{\circ ,7_t\},\{7_h,8_t\},\{8_h,\circ \}\big \} \big \} \end{aligned}$$

is given in Fig. 1. It is easy to confirm that the adjacency and cross edges each form a matching, so that each connected component of the graph will be either a cycle or a path. Note that connected components of the graph are only loosely related to the chromosomes; connected components can span multiple chromosomes.

We denote a cross edge by the label of the vertices that they connect. We denote the connected components of the graph by the set of cross edges that comprise them. The connected components of the graph in Fig. 1 are $\{5_t,4_h,6_h\}$, $\{5_h,6_t,2_t,1_h,\}$, $\{1_t,2_h,3_t,7_t\}$, $\{8_t,7_h\}$, and $\{3_h,4_t,8_h\}$. The *length* of a path or a cycle is the number of cross edges it has.

**Fig. 2:**
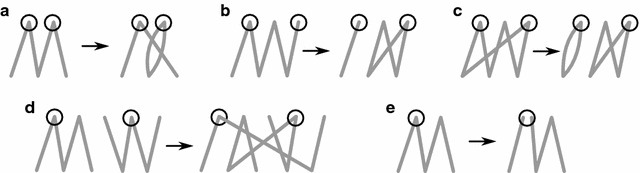
All possible DCJs that move one genome closer to the other. Adjacency edges are contracted, so that only the cross edges are shown in the connected components. Endpoints that are affected by the DCJ are *circled*. In the top row, extracting a cycle from (**a**) an even-length path, (**b**) an odd-length path, and (**c**) a cycle are depicted. Even-length paths can be combined to form two odd-length paths if one of the paths has endpoints in genome *A* and the other in genome *B*, as depicted in (**d**). An even-length path can be split into two odd length paths if the split is done in the genome with fewer vertices in the path, as depicted in (**e**)

To find sorting DCJs, we categorize the connected components by length. In Fig. 1 there is one cycle, two even-length paths, and two odd-length paths. The formula for the DCJ distance is

1 $$\begin{aligned} d_{DCJ}(A,B) = N - (C + I/2) \end{aligned}$$

where *N* is the number of blocks, *C* is the number of cycles, and *I* is the number of odd-length paths in *G*(*A*, *B*) [4]. Figure 2 depicts a comprehensive list of the possible sorting DCJs on an adjacency graph, and describes the conditions under which they may be applied. See Proposition 1 of [17] for a more thorough treatment. *G*(*A*, *A*), for some genome *A*, will always have 2*M* paths of length one and $N - M$ cycles of length two, where *M* is the number of chromosomes and *N* is the number of blocks.

### The minimum weighted rearrangements problem

Consider a genome $A_i$ made of a set of linear or circular chromosomes. Each rearrangement on this genome may have a certain likelihood of occurring. In the “Locality and the adjacency graph” section we will describe a DCJ move on $G(A_i,B)$ as a reconnection of two adjacency edges of $G(A_i,B)$; the resulting graph $G(A_{i+1},B)$ is identical to $G(A_i,B)$ aside from the connectivity of two adjacency edges. Therefore there is a bijection between edges of $G(A_i,B)$ and edges of $G(A_{i+1},B)$, so we can weight all pairs of genome adjacencies occurring in a sorting scenario by weighting all pairs of adjacency edges in *G*(*A*, *B*). For the set *P* of all pairs of adjacency edges in genome *A*, the weight function for a pair is $w:P \mapsto \mathbb {R} _+$, where $\mathbb {R} _+$ denotes the non-negative real numbers. The higher the value of *w* the less likely the rearrangement is to occur, e.g., a value of 0 represents a most likely rearrangement.

A sequence of rearrangements $\rho _1,\rho _2,\ldots ,\rho _d$ such that $(\cdots ((A\rho _1)\rho _2)\cdots \rho _d) = B$ is called a *sorting scenario*. The weight of a scenario is the sum of the weights of all the rearrangements in the scenario, i.e., $\sum _{i=1}^d{w(\rho _i)}$. The Minimum Weighted Rearrangements problem is the following.

#### **Problem 1**

Minimum Weighted Rearrangements

- **INPUT:** Genomes *A* and *B* and a weight function *w*.
- **OUTPUT:** A scenario of rearrangements turning *A* into *B*.
- **MEASURE:** The weight of the scenario.

### Positional constraints as colored adjacencies

Although chromosomes are represented as linear or circular sequences of syntenic blocks, in reality they correspond to molecules whose conformation within the nucleus is complex. Recent technological advances, called Hi-C, allow the mapping of chromosome conformation in various cell types and species [22–26]. The positional constraints introduced here are based on the principle that rearrangements (DCJ moves) involving pairs of adjacencies that are close in 3D space are more frequent than others. This model is supported by the pioneering work of Véron et al.  [27], who showed that loci that are distant in the linear ordering of the human chromosome yet close in the ordering of the mouse chromosome, are physically close (in 3D) in the human chromosome. Recently we have conducted a study on rearrangement scenarios showing that breakpoint pairs comprising a rearrangement are closer than expected by chance for intrachromosomal and interchromosomal rearrangements. This is true for multiple cell types from multiple laboratories [28]. In this paper, we use the observation that many moves are local to constrain the rearrangement scenarios that we compute. We call this the *positional* constraint.

**Fig. 3:**
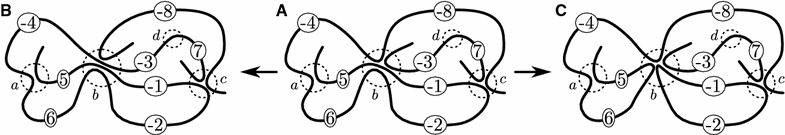
**A** A 2D cartoon of a possible 3D configuration for genome *A*. Adjacencies between syntenic blocks are classified by physically close regions, which are marked by *dashed circles* and labeled by the *alphabet* $\{a,b,c,d\}$. **B** Genome *A* after a reciprocal translocation has occurred at position *b*. **C** Genome *A* after an excision has occurred at position *b*

We incorporate the constraint by grouping adjacencies of the genome into classes that are more likely to swap endpoints. This idea is illustrated in Fig. 3, where the physical (3D) structure of genome *A* is drawn and the adjacencies are grouped into colored *localities*. According to Véron et al.  [27] and our recent results [28], rearrangements are more likely to occur between adjacencies at the same position.

### Locality and the adjacency graph

**Fig. 4:**
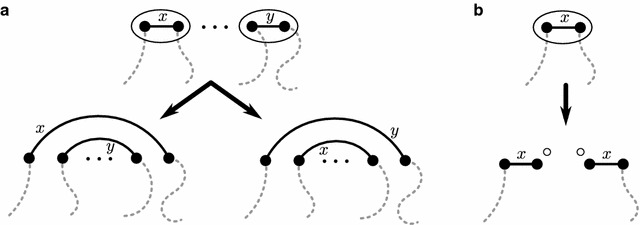
The update of colors by a DCJ. **a** Adjacency edges with *colors* *x* and *y* are reconfigured in two different ways for the same DCJ operation. In this case the reconfigurations are achieved by swapping either both right-hand endpoints or both left-hand endpoints of the adjacency edges. **b** The adjacency edge with *color* *x* is split to make two adjacencies of *color* *x* with two new telomeres

Each adjacency edge in *G* corresponds to an adjacency in genome *A* or *B*. The color of an adjacency is given to the adjacency edge it corresponds to. Figure 1 shows a coloring for the adjacencies of genome *A* that matches the localities in Fig. 3. The application of a DCJ operation to a genome has the effect of swapping the endpoints of two adjacency edges, or splitting an adjacency edge as in the case of Fig. 4e.

Throughout a DCJ sorting scenario, adjacency edges always keep the same color. Thus, each DCJ operation corresponds to one of two possible updates of the same pair of adjacency edges, as depicted in Fig. 4a.

### A positional weight function

Categorize rearrangements into two sets: those that are likely, and those that are not. Such a categorization of rearrangements is powerful enough to encapsulate the positional property discussed earlier.

A DCJ $\rho$ acts on one or two adjacencies. Our model labels each adjacency with some *color* from an alphabet $\Sigma$, and weights a DCJ based on the colors that are acted upon. Call $i_\rho$ and $j_\rho$ the adjacencies affected by $\rho$; $i_\rho = j_\rho$ if the DCJ acts on only a single adjacency, e.g., case (e) in Fig. 2. The color of an adjacency $i_\rho$ is written $col(i_\rho )$. Given a DCJ $\rho$, our weight function is

$$\begin{aligned} w(\rho ) = \left\{ \begin{array}{ll} 0 &{} \text {if}\,\, i_\rho = j_\rho \,\, \mathrm{or}\,\, col(i_\rho ) = col(j_\rho )\\ 1 &{} \text {otherwise.}\\ \end{array} \right. \end{aligned}$$

We call those DCJ moves that have zero weight *likely*, while we call all others *rare*. It is trivial to evaluate our weight function for a given DCJ; simply check the colors of the two adjacency edges that are affected.

Two restricted versions of the general problem are now described. The problem Minimum Local Scenario is exactly Minimum Weighted Rearrangements with the positional weight function *w*.

#### **Problem 2**

(MLS ) Minimum Local Scenario

- **INPUT:** Genomes *A* and *B* and positional weight function *w*.
- **OUTPUT:** A scenario of rearrangements turning *A* into *B*.
- **MEASURE:** The weight of the scenario.

The problem Minimum Local Parsimonious Scenario introduces the constraint that the scenario output is also a parsimonious scenario, i.e., a scenario of minimum length.

#### **Problem 3**

(MLPS ) Minimum Local Parsimonious Scenario

- **INPUT:** Genomes *A* and *B* and positional weight function *w*.
- **OUTPUT:** A parsimonious scenario of rearrangements turning *A* into *B*.
- **MEASURE:** The weight of the scenario.

## Minimum local parsimonious scenario

Since a solution to Minimum Local Parsimonious Scenario is limited to sorting moves, most connected components of *G*(*A*, *B*, *col*) must be sorted independently of each other, the exception being for even-length paths; all but one DCJ in Fig. 2 act on a single connected component. We first give a method for computing the number of rare operations per connected component when no pair of even-length paths exist, as in Fig. 2d. We then show in the “Even-length paths” section how to solve the problem when such pairs exist.

### Colored partitions

Consider a connected component *C* of the graph *G*(*A*, *B*, *col*). If *C* is monochromatic, i.e., has adjacency edges of a single color, then the component can be sorted with likely DCJs according to the listed moves in Fig. 2; the move that operates on more than one component in Fig. 2d need not be used since each path can be split on its own with a local move, as in Fig. 2e. If *C* is polychromatic then DCJs must be performed to separate the colors, since a fully sorted genome has components that each have only a single colored adjacency edge in genome *A*.

**Fig. 5:**
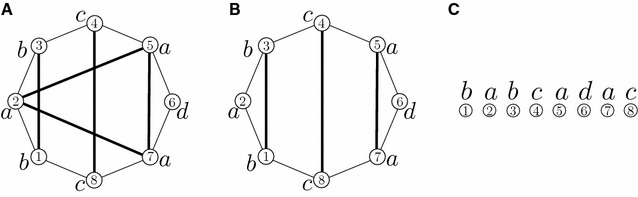
Colored partitions for the set [1, 8] where $col(1)=b$, $col(2)=a$, $col(3)=b$, $col(4)=c$, $col(5)=a$, $col(6)=d$, $col(7)=a$, and $col(8)=c$. Vertices are *circles* numbered by their order in the set [1, 8] and labeled by their *color*. *Thick black lines* are drawn between vertices that are in the same class of the partition. **A** The *crossing* partition $\{\{1,3\},\{2,5,7\},\{4,8\},\{6\}\}$. **B** The optimal *noncrossing* partition $\{\{1,3\},\{2\},\{4,8\},\{5,7\},\{6\}\}$.** C** The instance embedded on a line

Recall that $AA$-paths and $BB$-paths are paths that start and end in the same genome. In this subsection, we assume that there does not exist both an $AA$-path and a $BB$-path in the graph (Fig. 2d). Ouangraoua and Bergeron established that the DCJs in a sorting scenario can be done in any order for such a graph and that every component will be sorted independently, thereby defining a noncrossing partition on each component (see sections 3 and 4 of [17]). Later in this section we show that Minimum Local Parsimonious Scenario on a single component is equivalent to the following problem concerning a generalization of noncrossing partitions. A *partition* of a set is a collection of pairwise disjoint subsets whose union is the entire set. The subsets are called *classes*. [1, *n*] is the set of integers from 1 to *n*.

#### **Definition 1**

A noncrossing partition *is a partition*$\mathcal {P}$*of* [1, *n*] *such that for any classes*$S_i,S_j \in \mathcal {P}$*if we have*$p < q < p' < q'$*for*$p,p' \in S_i$*and*$q,q' \in S_j$*, then*$S_i = S_j$. *Anoncrossing colored partitionis a noncrossing partition where for any*$p,p' \in S_i$, $col(p) = col(p')$.

Another way to define a noncrossing partition is on a convex polygon. A noncrossing partition is a partition of the vertices of an *n*-gon with the property that if you draw a line between all pairs of vertices in the same class, for all classes, then no two lines from different classes intersect. A *colored partition* has colored vertices, and respects the property that any pair of vertices in the same class of the partition have the same color (see Fig. 5A, B).

#### **Problem 4**

(MNCP) Minimum Noncrossing Colored Partition

- **INPUT:** Set size *n*, color set $\Sigma$, and color function $col:[1,n] \rightarrow \Sigma$.
- **OUTPUT:** A noncrossing colored partition.
- **MEASURE:** The cardinality of the partition.

We present a polynomial-time algorithm for the Minimum Noncrossing Colored Partition problem, which according to Lemma 2 (later in this section) gives a solution to Minimum Local Parsimonious Scenario on a single component. We describe the algorithm on an instance that has been embedded on a line where the left-most vertex ① represents the smallest element of the set, as shown in Fig. 5C. For an interval [*i*, *j*], let *NCP*(*i*, *j*) be the number of classes in the MNCP on that subproblem. Thus, *NCP*(1, *n*) corresponds to the Minimum Noncrossing Colored Partition of [1, *n*].

For any interval [*i*, *j*] we have $NCP(i,i)=1$, and the following recurrence.

$$\begin{aligned} NCP(i,j) = \min \left\{ \begin{array}{ll} NCP(i,j-1)+1 &{} \text {for}\,\, i < j,\\ NCP(i,j-1) &{} \text {for}\,\, i < j \,\, \mathrm{and}\,\, col(i) = col(j)\\ NCP(i,k-1)+NCP(k,j) &{} \text {for all}\,\, k\,\, \mathrm{where}\,\, i < k < j\\ \end{array} \right. \end{aligned}$$

The first case corresponds to the creation of a new class with the single element *j*. The second case is applicable when element *j* is the same color as element *i*; in this case *i* and *j* become part of the same class, all the other classes staying the same. The third case tests combinations of subproblems; this case is pertinent when the $col(i) = col(k-1)$ or $col(k) = col(j)$. It is easy to confirm that any feasible solution to MNCP is scored by the recurrence. This dynamic program runs in $O(n^3)$ time.

We now show the link between MLPS and MNCP. Consider component *C* to be sorted. Pick an arbitrary vertex of *C* if it is a cycle, or either endpoint of *C* if it is a path, and consider an ordering of the vertices of genome *A* based on a traversal of the edges of *C* from that vertex. Embed the vertices of the component on a circle with respect to that ordering, and the edges so that they remain inside the circle. Call this a *circular embedding* of the component. Consider a sorting scenario for *C* that corresponds to a sequence of adjacency graphs $C_0,C_1,\ldots ,C_d$ ($C=C_0$). Call $C_i^{\circ }$ the graph $C_i$ with vertices embedded according to the circular embedding of $C_0$.

#### **Lemma 1**

[17] $C_i^{\circ }$*has no pair of crossing adjacency edges for any i*.

#### *Proof*

By construction, all adjacency edges in $C_0^{\circ }$ connect adjacent vertices on the circle, so none of them cross. Assume that $C_j^{\circ }$ has crossing adjacency edges and $C_{j-1}^{\circ }$ does not. This implies that the *j*th DCJ did not split a component. This is a contradiction since every sorting move on *C* splits a component, never creating both an $AA$-path and $BB$-path. $\square$

#### **Lemma 2**

*Given a connected component C*, *Minimum Local Parsimonious Scenario on C can be solved by Minimum Noncrossing Colored Partition*.

#### *Proof*

First, transform an instance of MLPS on a single component to an instance of MNCP. Given a cycle *C* representing genomes *A* and *B*, map the set of elements [1, *n*] from the set of adjacency edges of *A* ordered according to a circular embedding of *C*. The color function *col* maps each element to its corresponding adjacency edge’s color.

Now transform an optimal solution of MNCP into an optimal solution for MLPS. Clearly, any partition of [1, *n*] corresponds to a partition of adjacency edges of genome *A*. We show that there always exists a scenario of DCJs whose prefix separates *C* into connected components according to the partition. Any two edges of the same component can be chosen for a DCJ [17] and the DCJs on a cycle can be done in any order (Lemma 1). Since the ordering of the edges on the cycle corresponds to the ordering on [1, *n*], an edge partition of size *k* can be achieved with $k-1$ DCJs. Since *k* is minimum over all feasible partitions and the remaining DCJs of the scenario are likely, the constructed scenario has a minimum number of rare DCJs. $\square$

In fact, the two problems are equivalent. We omit the reduction in the other direction since it is out of the scope of this paper.

### Even-length paths

A Minimum Noncrossing Colored Partition can be computed in polynomial time for a single component independent of all others. Yet it is possible to mix components in a parsimonious DCJ scenario. As described in Fig. 2, the only parsimonious DCJs that *mix* components are those that act on one edge from an $AA$-path and one edge from a $BB$-path. Call *AA* (*BB* respectively) the set of $AA$-paths ($BB$-paths respectively) in the adjacency graph. The key observation is that once a path has been mixed with another, the result is always two odd-length paths which subsequently cannot be mixed with any other. Thus we devote this section to the computation of which pairs of paths $(p,q) \in AA \times BB$ will be mixed in an optimal solution, and which paths will remain unmixed.

Any pair (*p*, *q*) can be mixed in several ways. For all possible DCJs that mix them, we compute the MNCP on the resulting components. The minimum MNCP over all mixings is the cost in rare moves for mixing the two paths. To compute the pairs of paths to be mixed in an optimal solution, we use the inverse of these costs—the number of likely moves—as weights in a bipartite graph.

Take the elements of *AA* and *BB* as vertices in a complete bipartite graph, and label each edge (*p*, *q*) with the maximum number of likely DCJs for the mixing of paths *p* and *q*. Any even-length path could alternatively be used independently of any other, so there is a vertex $v'$ for each $v \in AA \cup BB$ with a single edge $(v,v')$ labeled by the number of likely moves on *v* alone (computed using the MNCP on that component). Algorithm 1 computes the minimum number of rare DCJs in a parsimonious scenario. It is easy to modify the algorithm to give the list of DCJs. 
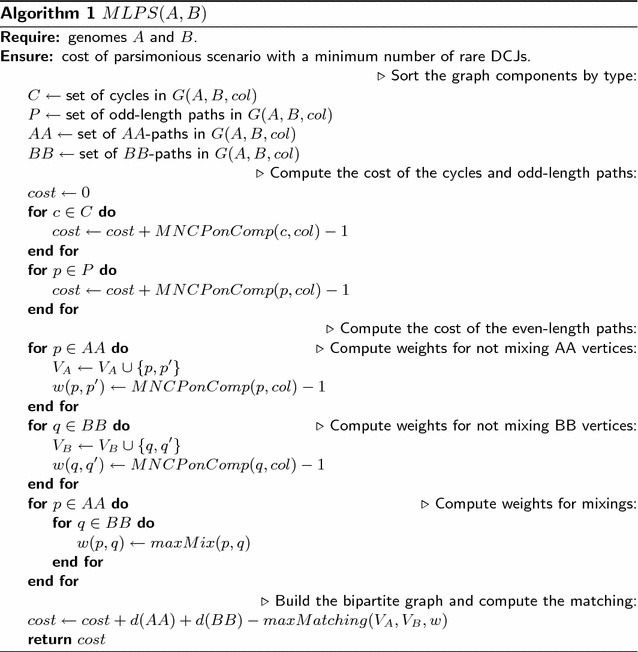


The function *MNCPonComp*(*c*, *col*) computes the Minimum Noncrossing Colored Partition on the given component *c*. In other words it builds the color function *col* according to the component *c* and then calls *MNCP*(1, *n*, *col*) where *n* is the number of adjacency edges on the *A* side of the component *c*. The function *maxMix*(*p*, *q*) computes the maximum number of likely DCJs over all possible DCJs that use one edge from *p* and one edge from *q*. The function *d*(*AA*) computes the sum of DCJ distances from each component in *AA* using Formula 1. The function $maxMatching(V_A,V_B,w)$ builds the bipartite graph with vertices $V_A$ on one side and vertices $V_B$ on the other, and the edges described by the weight function *w*.

To summarize, any path can be mixed at most once in a parsimonious scenario. Potential mixings, as well as potential non-mixings, are encoded into a bipartite graph with edges weighted by the cost of a mix. A maximum weight matching in this graph corresponds to a scenario that minimizes the number of rare moves on the paths. All other connected components of the graph are sorted using the Minimum Noncrossing Colored Partition on the component.

The running time of our algorithm is dominated by the weighting of the edges on the bipartite graph. Consider all mixings done between elements of *AA* and elements of *BB*. A particular adjacency edge *e* from a given path $p \in AA$ will take part in exactly one DCJ with every edge *f* from a path $q \in BB$ throughout the weighting process. Therefore for each pair (*e*, *f*), *e* being an edge from a path in *AA* and *f* being an edge from a path in *BB*, we will compute the MNCP on the resulting mix. If the number of edges in the paths *AA* (respectively *BB*) is *n*(*AA*) (respectively *n*(*BB*)), then the running time of our algorithm is $O(n(AA)n(BB)n^3)$. In the worst case, half of the edges are used in $AA$-paths and half in $BB$-paths, yielding a running time of $O(n^5)$.

### Faster mixing of even-length paths

In the previous section, edges of the bipartite graph are scored by the function *maxMix* that computes the maximum number of likely DCJs over all possible mixings of two paths. The analysis includes the multiplicative term *n*(*AA*)*n*(*BB*) reflecting the process of actually trying all possible mixings when labeling the edges of the bipartite graph. We now show how to mix paths more efficiently.

**Fig. 6:**
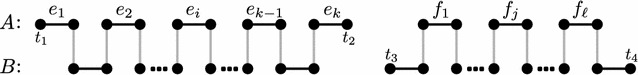
An $AA$-path and a $BB$-path

Define the *A-edges* of a component of the graph *G*(*A*, *B*) to be those edges connecting two nodes in genome *A*. Consider paths $p \in AA$ and $q \in BB$ where *p* is the path with *A*-edges $e_1,e_2,\ldots ,e_k$ and telomeres *t*1 and *t*2, and *q* is the path with *A*-edges $f_1,f_2,\ldots ,f_\ell$ and telomeres *t*3 and *t*4 (see Fig. 6). Construct two different cycles from *p* and *q*, cycle *c*1 results from joining *t*1 to *t*3 and *t*2 to *t*4 by cross edges, and cycle *c*2 results from joining *t*1 to *t*4 and *t*2 to *t*3. The *A*-edges of *p* can then be ordered circularly in *c*1 where edge $e_1$ follows edge $e_k$. Similarly, $f_1$ follows $f_\ell$ in *c*2. We show that there is a bijection between scenarios that start by mixing *p* and *q*, and scenarios that act on one of these two cycles by first performing a DCJ between an *e* edge and an *f* edge.

There is an obvious bijection between edges of $p \cup q$ and *c*1, and between edges of $p \cup q$ and *c*2. Consider the mix move acting on edge $e_i$ in *p* and $f_j$ in *q*. The result is either:

1. paths $e_1,e_2,\ldots ,\,e_i,f_{j-1},f_{j-2},\ldots ,f_1$ and $e_k,e_{k-1},\ldots ,\,e_{i+1},f_j,f_{j+1},\ldots ,f_\ell$, or
2. paths $e_1,e_2,\ldots ,\,e_{i-1},f_j,f_{j-1},\ldots ,f_1$ and $e_k,e_{k-1},\ldots ,\,e_i,f_{j+1},f_{j+2},\ldots ,f_\ell$, or
3. paths $e_1,e_2,\ldots ,\,e_i,f_{j+1},f_{j+2},\ldots ,f_\ell$ and $e_k,e_{k-1},\ldots ,\,e_{i+1},f_j,f_{j-1},\ldots ,f_1$, or
4. paths $$e_1,e_2,\ldots ,\,e_{i-1},f_j,f_{j+1},\ldots ,f_\ell$$ and $$e_k,e_{k-1},\ldots ,\,e_i,f_{j-1},f_{j-2},\ldots ,f_1$$.

The DCJ acting on $e_i$ and $f_j$ in *c*1 yields two cycles partitioning the edges as they are in either Case 1 or Case 2. The DCJ acting on $e_i$ and $f_j$ in *c*2 yields two cycles partitioning the edges as they are in either Case 3 or Case 4. Since odd length paths and cycles can only be sorted by cycle-extraction moves (see Fig. 2), each scenario mixing $e_i$ and $f_j$ maps to a scenario on *c*1 or *c*2. The bijection follows from the fact that moves on a cycle can be ordered in any way (Lemma 1).

Due to the bijection between mixing scenarios on *p* and *q*, and scenarios on *c*1 or *c*2, the MNCP by mixing *p* and *q* must be either the MNCP on *c*1 or the MNCP on *c*2. Thus, our algorithm to compute *maxMix*(*p*, *q*) returns the maximum of *MNCPonComp*(*c*1, *col*) or *MNCPonComp*(*c*2, *col*) or $MNCPonComp(p,col) + MNCPonComp(q,col)$.

Our new version of *maxMix* removes a linear factor from the overall computation time. Note $a_{1}, \dots , a_{x}$ the sizes of the paths in *AA* and $b_{1}, \dots , b_{y}$ the sizes of the paths in *BB* so that $|AA|=\sum _{i=0}^{x}a_{i}$ and $|BB|=\sum _{j=0}^{y}b_{j}$.

"Colored partitions" section shows that the number of steps required to solve MNCP on a component of size *m* is less than $c \times m^3$, for some constant *c*. For each pair of paths, we compute *MNCPonComp* three times, so the number of steps required to label all the edges of the complete bipartite graph is at most

$$\begin{aligned} 3c\sum _{i=0}^x\sum _{j=0}^y(a_i+b_j)^3&= 3c\sum _{i=0}^x\sum _{j=0}^y(a_i^3+b_j^3+3a_i^2b_j+3a_ib_j^2) \\&=3c\Big (y\sum _{i=0}^xa_i^3+x\sum _{j=0}^yb_j^3+3|BB|\sum _{i=0}^xa_i^2+3|AA|\sum _{j=0}^yb_j^2\Big ). \end{aligned}$$

The terms *y*, *x*, |*AA*|, and |*BB*| are clearly *O*(*n*). Since the largest terms $\sum _{i=0}^{x}a_{i}^3$ and $\sum _{j=0}^{y}b_{i}^3$ are in $O(n^3)$, the complexity of the bipartite graph labeling step is $O(n^4)$. Since sorting all non-even paths takes $O(n^3)$ time, our complete algorithm takes $O(n^4)$ time in the worst case.

## Conclusion

The number of parsimonious DCJ scenarios between two genomes is exponential in the distance between them. However, many of the scenarios are probably unrealistic in the biological sense. This paper takes a step towards modeling realistic scenarios by posing optimization problems that take into account positional constraints. An example of such a positional constraint is the 3D proximity of genome segments given by Hi-C experiments.

An $O(n^4)$ algorithm is proposed for computing a parsimonious DCJ scenario that is most likely, given an edge-coloring function that classifies DCJ as “likely” or “unlikely”. In practice the algorithm will be $O(n^3)$ since we expect long even-length paths to be rare in nature. For example, the adjacency graph for the mouse/human syntenic map built by Véron et al.  [27] from one-to-one orthologs in Biomart has only 182 edges in even-length paths out of a total of 13,302 edges. The largest connected component has 35 edges.

From a biological perspective, a solution to Minimum Local Parsimonious Scenario corresponds to finding a maximum likelihood scenario in a situation where likely and unlikely scenarios are both rare, and the difference between the likelihoods of likely and unlikely moves is not very large. In this situation, a most parsimonious scenario made of *k* unlikely moves is more likely than a non-parsimonious scenario made of $k+1$ likely moves. Thus the maximum likelihood scenario is the most parsimonious scenario that involves the smallest number of unlikely moves.

We introduce the Minimum Noncrossing Colored Partition problem—a generalization of the Maximum Independent Set problem on circle graphs—for weighting the edges of a bipartite graph, on which we obtain a maximum matching. While this technique is essential to our algorithm for finding DCJ scenarios, we believe it will also come in handy for an algorithm that finds likely *inversion* scenarios (e.g., for handling the infamous “hurdles”). A multitude of biologically relevant variations on this problem exist, including variations on the model of genome rearrangement, a variant where edges have multiple colors, and a bi-directional sorting variant where edges are weighted on both genomes according to the chromatin conformation on each. Models that incorporate uncertainty or evolution in the Hi-C data would also be relevant. We hope that this work provokes further study from both the algorithmic and the biological perspectives.
